# Acute attenuation of fatigue after sodium bicarbonate supplementation does not manifest into greater training adaptations after 10-weeks of resistance training exercise

**DOI:** 10.1371/journal.pone.0196677

**Published:** 2018-05-02

**Authors:** Jason C. Siegler, Paul W. M. Marshall, Harrison Finn, Rebecca Cross, Kurt Mudie

**Affiliations:** 1 Sport and Exercise Science, School of Science and Health, Western Sydney University, Sydney, Australia; 2 Neuroscience Research Australia, Sydney, Australia; 3 Institute of Sport, Exercise and Active Living (ISEAL), Victoria University, Melbourne, Australia; Garvan Institute of Medical Research, AUSTRALIA

## Abstract

**Purpose:**

In two concurrent studies, we aimed to a) confirm the acute effect of 0.3 g·kg^-1^ body weight (BW) sodium bicarbonate (NaHCO_3_) supplementation on central and peripheral mechanisms associated with explosive power (**Study 1**) and b) determine whether chronic NaHCO_3_ supplementation would improve the adaptive response of the neuromuscular system during a 10-week resistance training program (**Study 2**).

**Methods:**

Eight resistance trained participants volunteered after providing written consent. The experimental design consisted of a week of baseline testing, followed by ten weeks of training with progress measures performed in Week 5. **Study 1** involved neuromuscular measurements before and after the leg extension portion of a power based training session performed in Week 1. Changes in maximal torque (MVT) and rates of torque development (RTD), along with other variables derived from femoral nerve stimulation (e.g. voluntary activation, neural recruitment) were analysed to determine the extent of fatigue under NaHCO_3_ or placebo conditions. Changes in these same variables, coupled with functional 1-repetition maximum leg extension strength, were measured in **Study 2** from baseline (Week 0) to Week 5, and again at Week 10.

**Results and conclusion:**

In **Study 1**, we observed a decline after the leg extension task in both MVT (~ 30%) and rates of torque production (RTD) irrespective of acid-base status, however the decline in maximal RTD (RTD_MAX_) was nearly 20% less in the NaHCO_3_ condition when compared to placebo (mean difference of 294.8 ± 133.4 Nm·s^-1^ (95% CI -583.1 to -6.5 Nm, p < 0.05)). The primary finding in **Study 2**, however, suggests that introducing NaHCO_3_ repeatedly during a 10-week RT program does not confer any additional benefit to the mechanisms (and subsequent adaptive processes) related to explosive power production.

## Introduction

Resistance training (RT) is an integral component of conditioning programs throughout many, if not all, athletic and sporting codes. Most RT programs emphasise strength and/or power development in an attempt to improve various aspects of the skeletal muscle and nervous systems. The degree to which these integrated, or neuromuscular, systems respond to an RT program will be influenced by a multitude of factors (e.g. individual morphology, training experience, programming & scheduling, nutrition, etc. [1–3]), and is often highly individual. The efficacy of manipulating certain training variables (i.e. load, contraction velocity) to achieve specific neuromuscular adaptations can also be influenced through the timing and implementation of nutritional interventions or the use of ergogenic aids. For example, ingestion of ergogenic aids such as creatine and caffeine have repeatedly demonstrated capacity for acutely improving explosive muscular power [4–6]. Sodium bicarbonate, another supplement widely purported to improve anaerobic exercise performance and sustain muscular power, may also be a candidate but to our knowledge has not been investigated in a prolonged (i.e. > single session) RT context.

The efficacy of sodium bicarbonate (NaHCO_3_) supplementation to improve sport performance has been researched since the 1970’s [7,8], and is common practice amongst certain elite athlete populations (e.g. track sprint cycling, rowing). Briefly, doses between 0.2–0.3 g·kg^-1^ body weight (BW) lead to a ~ 5 to 6 mmol·L^-1^ increase in blood bicarbonate, which is believed to facilitate the removal of unbuffered intracellular protons (H^+^) commensurate with intense exercise [9,10]. As such, it has predominately been used in the context of exercise constituting high metabolic demand, where glycolysis (and consequently high levels of H^+^ production) is the prevailing pathway for energy provision. However, recent research suggests sodium bicarbonate may also affect rapid, explosive force production [11,12]. Evidence in both animal and human exercise models have demonstrated that the muscles ability to rapidly generate force may be better maintained during fatiguing exercise after the application of NaHCO_3_ [11,12]. Given this body of evidence suggesting that NaHCO_3_ supplementation can maintain rapid force production during intense exercise, we believed an investigation into the use of NaHCO_3_ within an RT program was warranted.

Sodium bicarbonate (NaHCO_3_) supplementation has been examined in a single session RT context with equivocal results. Both Webster [13] and Portington [14] used four sets of leg extension exercises at a set percentage of 1-repetition maximum (RM) and measured repetitions completed on a 5^th^ set, with neither study observing an effect of NaHCO_3_. More recently, Carr [15] and Duncan [16] have reported increased volumes of exercise after NaHCO_3_ supplementation during whole-body exercise protocols (e.g. back squat + leg press + bench press). Respectfully, these studies only used gross measures of muscle function to assess the efficacy of NaHCO_3_, and therefore would not have had the sensitivity to detect subtle and acute changes in explosive force production. Furthermore, none of these studies examined the training adaptation after a period of combined RT and NaHCO_3_ supplementation. We believe the limited number of studies, the inherent variability and lack of sensitivity of performance measures used, and absence of training related outcomes has not adequately addressed the question as to whether or not NaHCO_3_ supplementation may be appropriate to embed within an RT program.

Therefore, we aimed to address this gap in the literature by conducting two studies. The first study aimed to confirm the acute effect of NaHCO_3_ supplementation on fatiguing, repeated knee extension exercise and to determine to what extent, if any, alkalosis has on central and peripheral mechanisms contributing to rapid force production. Given our recent findings regarding the attenuation in the decrement of rapid force production after NaHCO_3_ supplementation, we hypothesised that inducing alkalosis in the first study would result in a similar improvement after leg extension exercise. The aim of the second study was to investigate whether incorporating NaHCO_3_ supplementation into a 10-week RT program would improve the adaptive response of the neuromuscular system over non-supplemented training. We hypothesised that the acute benefits from NaHCO_3_ would manifest into greater long term/chronic training outcomes though improvements in mechanisms central to rapid force production in comparison to training alone.

## Materials and methods

### Participants

Six resistance trained men (mean ± SD; age 26 ± 5 years, height 1.81 ± 0.08 m, weight 90.4 ± 13.3 kg) and two resistance trained women (mean ± SD; age 25 ± 3 years, height 1.62 ± 0.06 m, weight 67.6 ± 3.4 kg) volunteered to participate in this study after providing informed written consent. All volunteers had at least 3-years resistance training experience (≥ 3 times per week for most training weeks of the year), and described regular performance of both upper and lower body resistance exercises. All participants were familiar with the primary quadriceps resistance exercise (i.e. leg extension), but none reported weekly performance of the movement prior to volunteering for this study. All procedures in this study were approved by the Western Sydney University Human Research Ethics Committee (H10839), and were conducted in accordance with the Declaration of Helsinki.

### Experimental design

**Fig 1:**
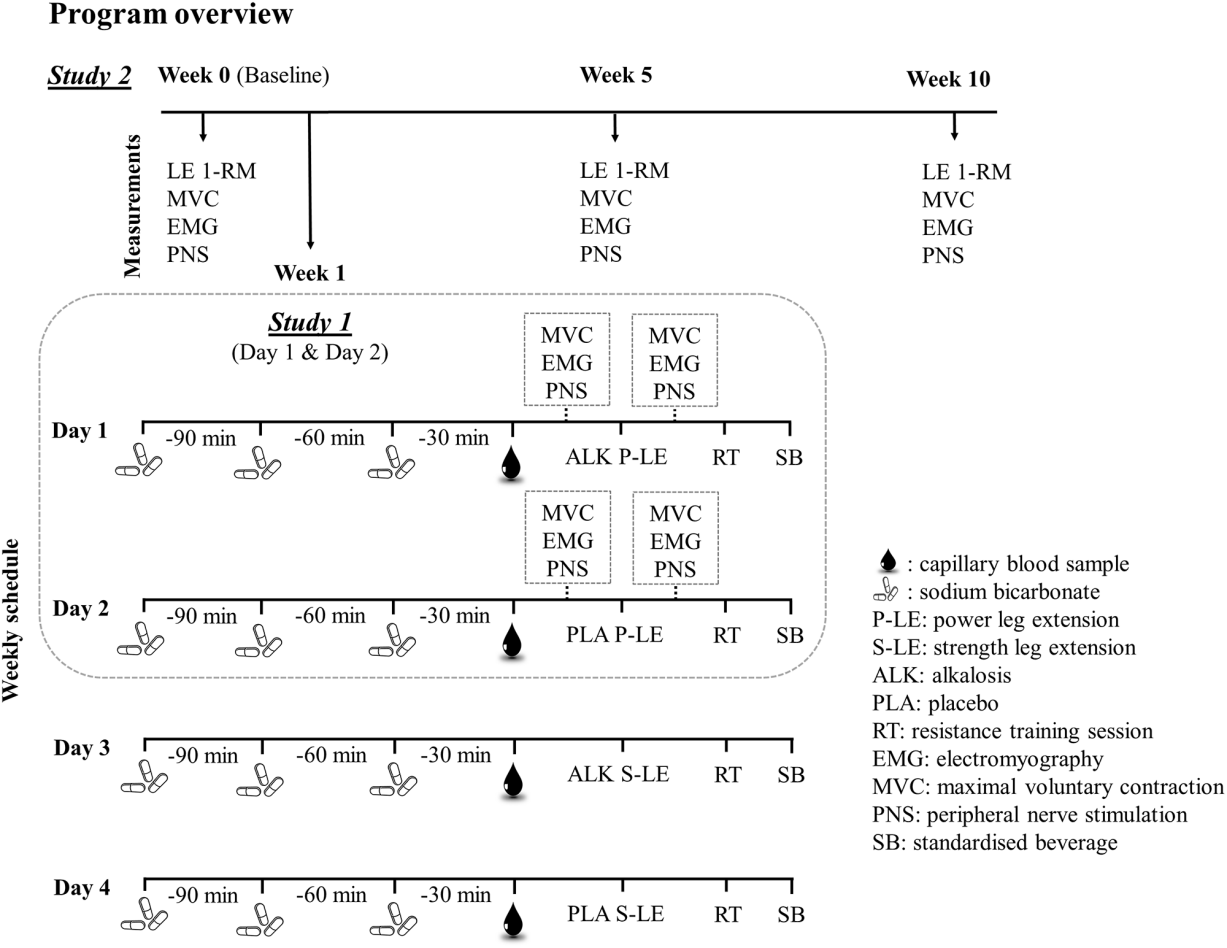
Schematic representation of Studies 1 and 2.

A schematic of **Studies 1 & 2** is provided in Fig 1. Both studies were conducted in a double-blind, randomized and counterbalanced manner. The experimental design consisted of a week of baseline testing (Week 0), followed by ten weeks of training with progress measures performed in Week 5, and at the conclusion of the training program in Week 10. **Study 1** (acute) involved laboratory measurements performed before and after the leg extension portion of the power based training sessions (*see Training Program*) performed in Week 1 to examine the acute response to training with or without supplementation. **Study 2** (chronic) involved reporting of training outcomes measured at Week 0, Week 5 and Week 10. There was no supplementation during these measurements.

In **Study 2**, each leg of a participant was randomly assigned to either receive NaHCO_3_ supplementation or placebo for the duration of the 10-week training period. Randomization and administration of the supplements prior to each training session was performed by a researcher who had no involvement with participant recruitment, testing, training or data analysis. Participants’ legs were randomly assigned in blocks of four with equal numbers of each leg (right/left) assigned to each condition (Fig 1). All training sessions were directly supervised 1:1 by an experienced exercise physiologist who was blinded to leg allocation and was not involved with any laboratory testing.

### Training program

For the quadriceps, a daily undulating periodization model was used where each leg (ALK or PLA) was trained twice per week, with one session being a power based session and the other a strength session (training week example, ALK-power, PLA-power, ALK-strength, PLA-strength (Fig 1)). The power session required participants to perform maximal effort leg extensions with a load corresponding to 40% of 1-RM. Participants performed continuous maximal efforts for 30 s with 30 s rest, repeated for five bouts. If a participant failed during the 30 s ‘work’ period, a 3 s rest was provided before another repetition was attempted. For the strength session, working leg extension sets (1.5 to 2-min inter-set rest intervals) were mainly prescribed to avoid repetition failure, thus allowing accrual of repetitions across a range of high-intensity contractions above 80% 1-RM. The primary working sets included 10 repetitions (reps) x 60% 1-RM, 2 sets x 5 reps at 80% 1-RM, 1 set x 5 reps at 85% 1-RM, 1 set x 3 reps at 87.5% 1-RM, and 1 set x 2 reps at 90% 1-RM. Finally, participants were required to perform two sets to repetition failure (inability to reach minimum range of motion) at 80% 1-RM. All repetitions in the strength session were performed as explosively as possible in the concentric phase, with a controlled eccentric lowering of 1.5 to 2 s. The leg extension prescription was rotated from week to week, and the sequence of sessions was randomly assigned between participants to avoid order effects influencing training outcomes.

The remainder of the training program, with the leg extension component (*and experimental measurements if applicable*) performed at the start of every session, was designed to train each major muscle group twice per week with a daily undulating periodization and no other exercise prescribed for the quadriceps. The primary training days remained consistent throughout the training program, with the only week-to-week variation being the leg extension component at the start of each session. All exercises were prescribed with a repetition-maximum scheme. If the required repetition range was not met (for the first set only) the load was adjusted for subsequent sets and training session. If required repetition levels were not met after the first set, no changes to load were made.

### Supplementation

All participants were provided with a training log that detailed both their exercise program and instructions for supplementation and post-exercise nutrition. Before every training session participants were required to consume their supplement (placebo (PLA): 0.3 g·kg^-1^BW calcium carbonate or alkalosis (ALK): 0.3 g·kg^-1^BW NaHCO_3_), which was equally divided into three separate doses. Capsules were consumed at -90, -60, and -30 mins prior to the training session. To ensure compliance, capillary blood was collected in duplicate immediately prior to training in a balanced heparinised 200μl blood gas capillary tube for analysis of acid-base status (pH, bicarbonate (HCO_3_^-^), and base excess (BE)) using a clinical blood gas analyser (ABL 800 basic series; Radiometer, Copenhagen, DK). After every workout participants were provided a standardized recovery drink to facilitate muscle protein synthesis including 0.5 g·kg^-1^BW maltodextrin and 0.4 g·kg^-1^BW whey protein isolate (Bulk Nutrients, Grove, AU), and were given their supplement capsules for the following days training.

### Experimental measurements

#### Functional strength & power

For **Study 2**, leg extension 1-RMs were measured on a Hammer strength iso-lateral leg extension (Life Fitness, Chatswood, AU). After a standard warm-up for 1-RM testing [17], participants attempted a maximal leg extension to a range of motion visually determined by the research assistant where the tibia was approximately parallel to the floor. The 1-RM was determined from the last successful lift before failure. Load increments for 1-RM testing were progressed in 2.5 kg amounts.

Laboratory testing of maximal isometric voluntary torque (MVT, Nm), rate of torque development (RTD, Nm.s^-1^), central motor output and quadriceps contractile function were performed using an isokinetic dynamometer (KinCom 125, Version 5.32, Chattanooga, US). Participants were seated with their hip and knee joints flexed to 90° and 75°, respectively. The centre of rotation of the lever arm was carefully aligned with the sagittal plane axis of the knee joint, and the lever arm of the dynamometer was firmly attached 2–3 cm superior to the lateral malleolus. The participant was firmly strapped into the chair with straps across the trunk during all testing procedures. Torque output signals were continuously sampled from the dynamometer at 1000 Hz (Powerlab, ADI Instruments, Sydney, AU), and low pass filtered at 10 Hz. Torque signals were calibrated in the resting test position for each participant’s limb weight (after all straps were applied), and a pre-determined calibration factor was applied to obtained signals for conversion of the recorded voltage to torque (Nm).

For measurement of MVT participants were required to push as fast and forcefully as possible for 3 to 5 s (warm-ups involved two efforts at 25%, 50%, and 75% perceived maximum). Four maximal efforts were performed before and after the training program, with femoral nerve stimulation applied during, and 3 to 4 s after each contraction to elicit the quadriceps potentiated twitch (PT).

#### Electromyography

Surface electromyograms (sEMG) were recorded from the vastus lateralis (VL) and vastus medialis (VM) using pairs of Ag/AgCl surface electrodes (Maxsensor, Medimax Global, Scorsby, AU). Electrodes (10 mm contact diameter, 10 mm inter-electrode distance) were placed in bipolar configuration parallel to the direction of the muscle fibres after careful skin preparation (shaving excess hair, careful abrasion with fine sandpaper and cleaning the skin with isopropyl alcohol swabs) approximately one hour prior to commencement of the training session. The inferior VL electrode was placed 8–12 cm superior to the lateral aspect of the patella, and the inferior VM electrode 3–4 cm superior to the medial aspect of the patella. Placements were recorded for each participant to ensure consistency between sessions. The reference electrode was placed on the medial tibial condyle. To minimise movement artefact, electrode cables were fastened to the side of the chair during testing. Surface electromyography (sEMG) signals were recorded using the ML138 Octal BioAmp (common mode rejection ratio > 85 dB at 50 Hz, input impedance 200 MΩ) with 16-bit analog-to-digital conversion, sampled at 4,000 Hz (ADI instruments, Sydney, AU). Raw signals were filtered with a fourth-order Bessel filter between 20 and 500 Hz.

#### Femoral nerve stimulation

A 5 x 9 cm custom electrode of aluminium foil and conduction gel was taped to the lateral aspect of the hip, between the iliac crest and greater trochanter as the anode. To identify nerve location for cathodal stimulation of the femoral nerve, a rubber insulated portable cathodal probe was used. The probe was moved around the femoral triangle using a single stimulus intensity of 30 mA until the largest muscle compound action potential (M-wave) was elicited from both the VL and VM recording sites. When optimal nerve location was identified, this was marked with a felt-tip pen and a 2 cm diameter Ag/AgCl surface electrode was applied.

The quadriceps were stimulated during maximal contractions by supramaximal doublets applied to the femoral nerve (1 ms square pulses) at 10 Hz by a high voltage (400 V) constant current stimulator (Digitimer DS7AH; Digitimer, Hertfordshire, UK). Stimulation intensity to be used during testing was determined by progressively increasing the current in 10 mA increments until plateaus occurred in twitch amplitude and M-waves in response to 10 Hz doublet stimulation. Supramaximal stimulation was ensured by increasing the final intensity from the plateau by 30% (intensity range for testing 80 to 190 mA). During each maximal effort, a superimposed doublet (10 Hz) was applied to the femoral nerve when torque had reached a visible plateau. Quadriceps resting potentiated twitches were then produced by delivering a doublet to the resting muscle, with the stimulation delivered 2 to 3 s post contraction.

#### Data processing

Contraction onset for voluntary torque and resting evoked twitches was identified with an automated algorithm in the software as the point after which torque exceeded the baseline by 2.5 Nm and 1 Nm, respectively. Vastus lateralis (VL) and VM muscle onsets were visually determined [18]. Torque recordings were used to analyse 1) the maximal voluntary torque recorded during the contraction up to the first point of stimulation (MVT, Nm), 2) rate of voluntary torque development (vRTD) was calculated as the average slope of the torque-time curve (Δtorque/Δtime) during the time periods 0–25 ms (vRTD_25_), 0–50 ms (vRTD_50_) and 0–100 ms (vRTD_100_) post contraction onset, and 3) maximum voluntary RTD (vRTD_MAX_) was calculated as the greatest average 10 ms slope throughout the first 200 ms of the contraction.

Voluntary activation (VA) was estimated from the 10 Hz stimulation using the superimposed twitch technique [19] according to the following formula [20]: VA (%) = 100 –(D * (T_sup_ / MVT) / PT) * 100, where D is the difference between the torque level just before the superimposed twitch (T_sup_) and the maximum torque recorded during the twitch, MVT is maximal voluntary torque during the entire contraction (not including the twitch response), and PT is the maximal amplitude of the resting potentiated twitch. In addition to the maximal amplitude of the resting potentiated twitch, the following variables were calculated from PT: 1) the time-to-peak twitch (TPT) and 2) the half relaxation time (^1/2^RT), calculated as the time from the peak amplitude until 50% of the maximal amplitude had been reached.

All sEMG variables during maximal contractions were normalised to the first respective M-waves elicited during 10 Hz stimulation applied to each contraction for data analysis (EMG/M, %). Surface electromyography (sEMG) recordings were used to analyse the following variables from each MVT measurement: 1) the electrically evoked M-wave from the 10 Hz doublet, calculated from the peak-to-peak amplitude of the VL and VM sEMG raw signal elicited during contraction, 2) the maximal amplitude of the VL (VL_MAX_) and VM (VM_MAX_) sEMG signal during MVTs based on processing the greatest average 250 ms root-mean-square (RMS) value, 3) the rate of sEMG rise for VL and VM calculated from the average slope of the RMS sEMG-time curve during the same time-periods as RTD processing post contraction onset, and 4) the maximal rate of sEMG rise for VL (VL_RERmax_) and VM (VM_RERmax_) calculated from the greatest 10 ms slope of the RMS EMG-time curve throughout the first 200 ms of the contraction.

#### Statistical analyses

All statistical analyses were completed using IBM SPSS Statistics version 22 (SPSS Inc., Chicago, USA). The Shapiro-Wilk Normality Test was applied to assess normality of distribution. The descriptive data are presented as mean ± SD. In **Study 1** (acute), pre-training blood acid-base data (pH, HCO_3_^-^ and BE) were analysed using student’s t test. Changes between pre- and post leg extension training in MVT, RTD variables, VA, VL_MAX_ and VM_MAX_, VL_RER_ and VM_RER_ variables, and RT variables were analysed using repeated measures ANOVA, with pre-training measures used as a covariate to control for differences in leg strength between dominant and non-dominant legs. In **Study 2** (chronic), blood acid-base data was analysed (repeated measures ANOVA) collectively from Week’s 1 to 4 and again from Week’s 6 to 9 to ensure appropriate pre-training acid-base status was achieved after supplementation. Changes in the performance measures (same as Study 1) between Week 0, Week 5 and Week 10 were analysed using a two-way mixed model (between and within-subjects) repeated measures ANOVA. In the event of a significant interaction, post hoc comparisons were made using a Bonferroni correction. Mean differences and standard error (SEM) between conditions and 95% confidence intervals (CI) were calculated when significant changes over time, or when differences between conditions were observed. Two-tailed statistical significance was accepted at p < 0.05.

## Study 1 results

### Blood acid-base

Whole blood acid-base findings were consistent with induced states of metabolic alkalosis in the ALK trial, where pH (ALK: 7.46 ± 0.02; PLA: 7.42 ± 0.02; p < 0.01), HCO_3_^-^ mmol·L^-1^ (ALK: 28.1 ± 1.1; PLA: 24.9 ± 1.0; p < 0.001) and BE meq·L^-1^ (ALK: 3.9 ± 1.5; PLA: 0.7 ± 1.6; p < 0.01) all differed from PLA.

### Leg extension session

**Fig 2:**
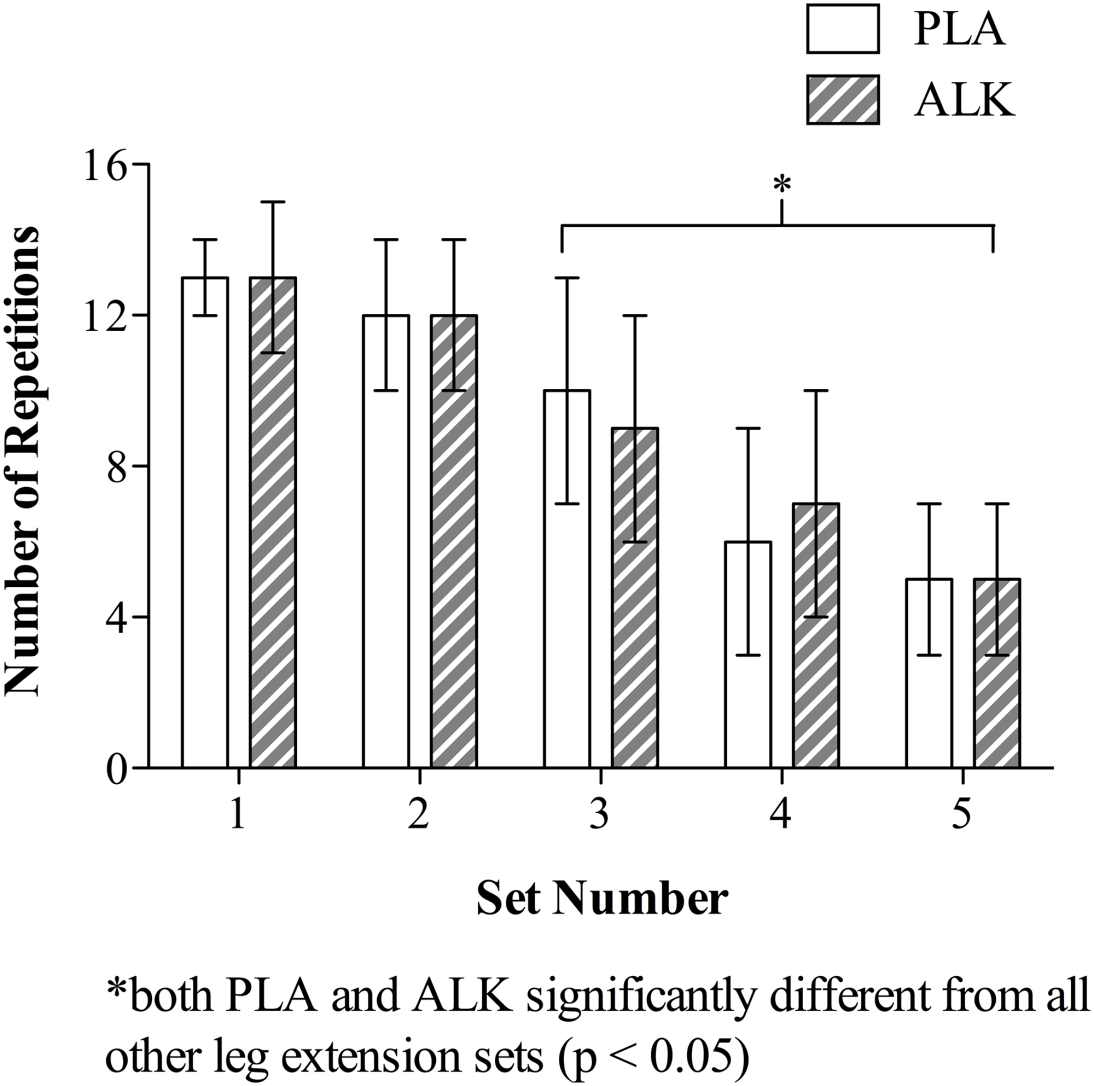
Number of repetitions completed during the five leg extension sets in Study 1. (ALK) alkalosis, (PLA) placebo, data are mean ± SEM.

The number of leg extension repetitions declined over the 5 sets (mean decline of 8.8 ± 0.8 reps from set 1 to set 5 (95% CI 11.9 to 5.6; p < 0.001; η^2^ = 0.89; Fig 2) irrespective of condition (ALK or PLA; p = 1.0).

### Maximal voluntary torque production

**Fig 3:**
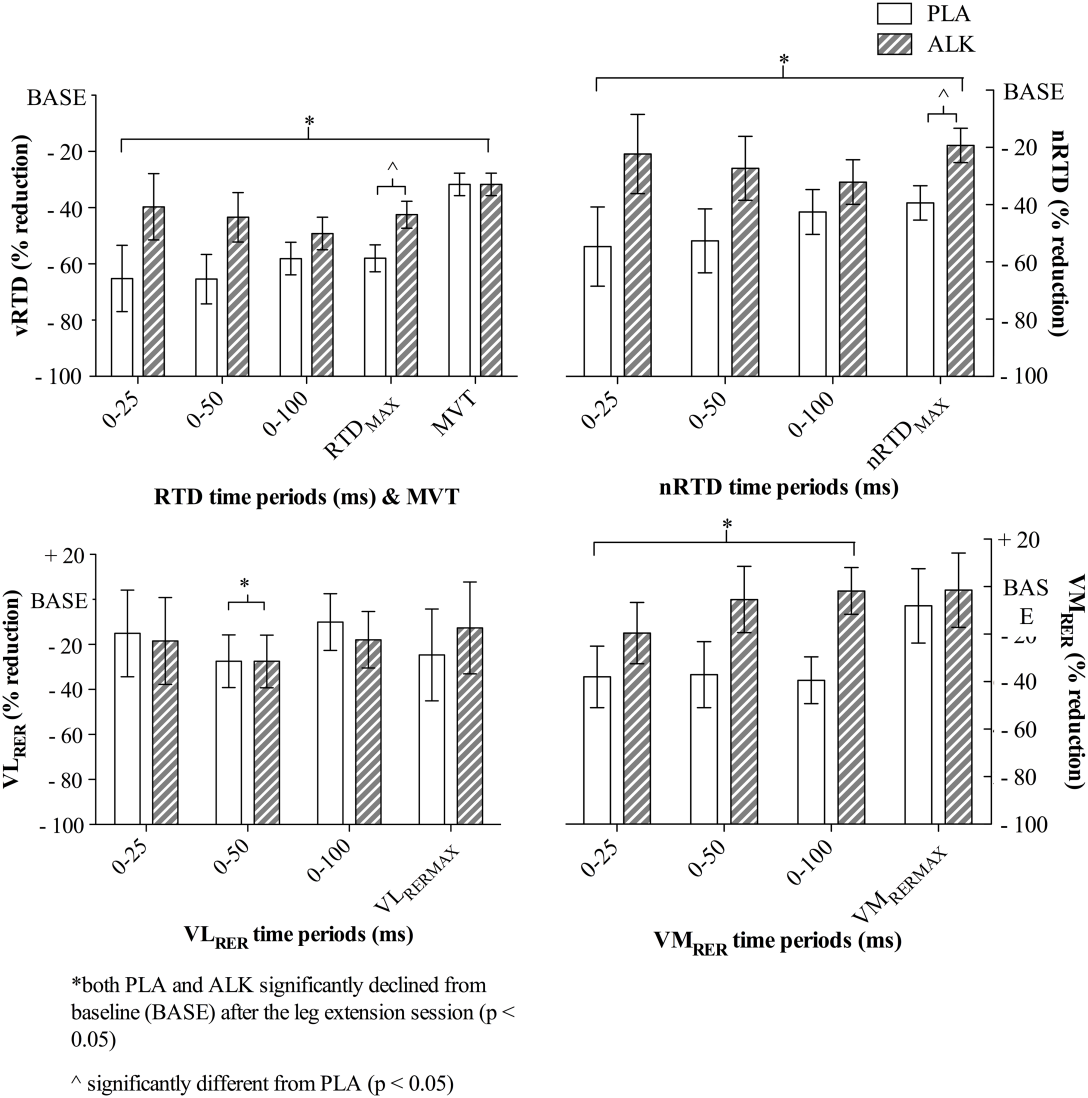
Percentage change (%) determined from measurements taken at rest (BASE) and immediately after the leg extension session in Study 1. Voluntary rate of torque development (vRTD), rate of torque development normalised to the corresponding maximal voluntary contraction (nRTD), and rates of EMG rise for vastus lateralis (VL_RER_) and vastus medialis (VM_RER_) normalised to the superimposed M-wave (%); Figure includes the maximal rate and the time periods (0–25, 0–50 and 0–100 ms) associated with these measurements; (ALK) alkalosis, (PLA) placebo (PLA), data are mean ± SEM.

Maximal voluntary torque (MVT) declined from pre- to post leg extension session (mean decline of 84.4 ± 10.2 Nm (95% CI -64.5 to -106.2 Nm); p < 0.001; η^2^ = 0.52; Fig 3) but was not different between ALK or PLA (p = 0.99). Although a decline was observed for all vRTD and nRTD rates (0–25, 0–50, 0–100 ms) and maximal values (Fig 3), only vRTD_MAX_ and nRTD_MAX_ exhibited a condition effect where ALK was better preserved post leg extension session (vRTD_MAX_: mean difference of 294.8 ± 133.4 Nm·s^-1^ (95% CI -583.1 to -6.5 Nm), p < 0.05, η^2^ = 0.27); nRTD_MAX_: mean difference of -1.5 ± 0.6 (95% CI -2.8 to -0.1), p < 0.05, η^2^ = 0.30; Fig 3).

### Descending central drive

Voluntary activation (VA; %) was unchanged post leg extension session in either ALK or PLA (p = 0.81; Table 1). Neither VL_MAX_ (%) nor VM_MAX_ (%) changed post session (p > 0.53), rates of rise in VL (VL_RER_) and VM (VM_RER_) declined in most time periods (p < 0.05; Fig 3), but were no different between ALK and PLA (p > 0.12; Fig 3).

**Table 1:** Presented are voluntary activation (VA; %), the maximal amplitude of the peak resting twitch amplitude at (PT; Nm), time-to-peak twitch (TPT; ms) and half relaxation time (^1/2^RT; ms), respectively, from pre- to post leg extension training session for placebo (PLA) and alkalosis (ALK) conditions. Data are presented as mean ± SD.

	VA		PT		TPT		^1/2^RT	
	*PLA*	*ALK*	*PLA*	*ALK*	*PLA*	*ALK*	*PLA*	*ALK*
BASE	89.8 ± 5.9	88.0 ± 4.8	100.2 ± 19.3	95.2 ± 17.9	158.1 ± 6.2	155.8 ± 6.1	99.8 ± 49.5	99.5 ± 59.1
POST	87.4 ± 11.4	91.7 ± 8.4	37.8 ± 14.5*	35.4 ± 12.7*	156.6 ± 9.0	179.6 ± 56.3	114.6 ± 10.8	99.3 ± 19.6

*significantly different from before the leg extension session (BASE) (p < 0.001).

### Evoked contractile properties

Maximal resting potentiated twitch amplitude (PT) declined post leg extension session (mean decline of 61.1 ± 5.6 Nm (95% CI -49.3 to -73.0 Nm); p < 0.001; η^2^ = 0.91), but was not different between ALK and PLA (p = 0.75; Table 1). Irrespective of ALK or PLA, neither TPT nor ^1/2^RT changed from pre- to post session (p > 0.16; Table 1).

## Study 2 results

### Blood acid-base

**Fig 4:**
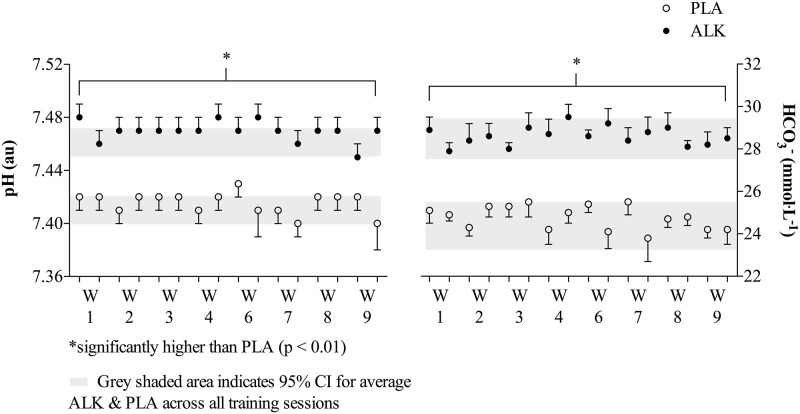
Pre-training blood pH and bicarbonate (HCO3-) values for each of the four training sessions throughout the 10-wk training program. Two alkalosis (ALK) and two placebo (PLA)) within the weekly training cycle (e.g. Wk 1, Wk 2, etc.), data are mean ± SEM.

A significant degree of alkalosis was present for all ALK training sessions when compared to PLA (pH: ALK 7.47 ± 0.02, PLA 7.42 ± 0.03, p < 0.001, η^2^ = 0.95; HCO_3_^-^ mmol·L^-1^: ALK 28.6 ± 1.6, PLA: 24.8 ± 1.7, p < 0.001, η^2^ = 0.94; BE meq·L^-1^: ALK 4.8 ± 2.0, PLA: 0.3 ± 2.2, p < 0.001, η^2^ = 0.94). There was no residual effect of the NaHCO_3_ supplementation during the training week, as the acid-base status pre-training each of the four training days was significantly different for all variables (p < 0.05; Fig 4).

### 1-RM leg extension

**Fig 5:**
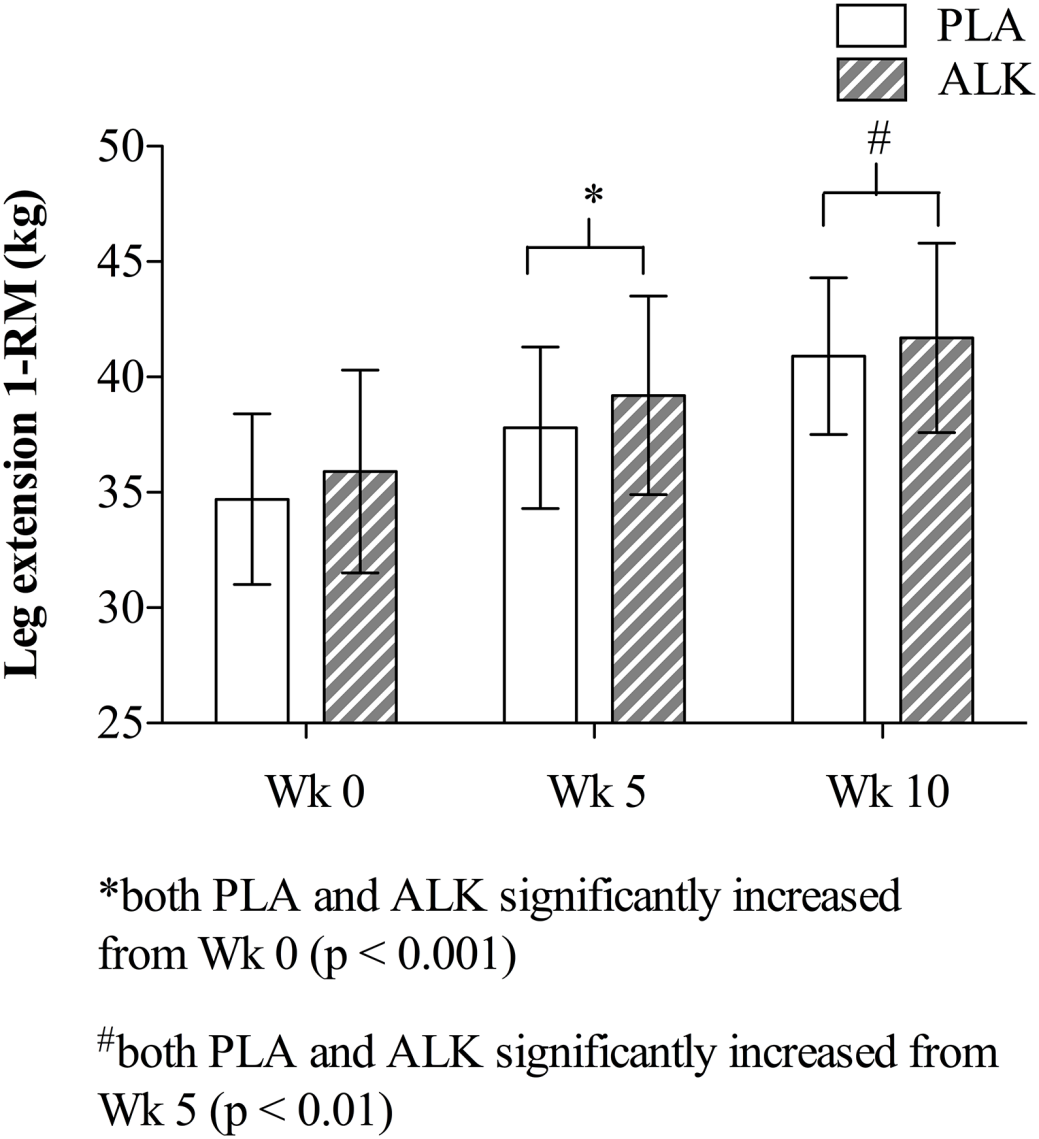
Leg extension one-repetition maximum (1-RM) at the beginning (W0), mid-point (W5) and end (W10) of the 10-wk training program. (ALK) alkalosis, (PLA) placebo, data are mean ± SEM.

One repetition maximum (1-RM) leg strength improved from Week 0 to Week 5 (mean increase of 3.2 ± 0.4 kg (95% CI 2.2 to 4.2 kg; p < 0.001; η^2^ = 0.87), and again from Week 5 to Week 10 (mean increase of 2.8 ± 0.7 kg (95% CI 0.9 to 4.7 kg; p < 0.001; η^2^ = 0.89). There was no influence of supplementation on the strength gains (p = 0.84; Fig 5).

### Maximal voluntary torque production

Maximal voluntary torque (MVT) did not change significantly over the 10-week period in either ALK (255 ± 72 to 275 ± 78 Nm) or PLA (269 ± 78 to 276 ± 76 Nm) (p > 0.14). Similarly, there were no significant changes across the training period for all vRTD and nRTD rates (0–25, 0–50, 0–100 ms) or maximal values (p > 0.11; Table 2).

**Table 2:** Presented are voluntary rate of torque development (vRTD; Nm·s^-1^) and normalised rate of torque development (nRTD) to maximal voluntary torque at the beginning (W0), mid-point (W5) and end (W10) of the 10-wk training program for placebo (PLA) and alkalosis (ALK) conditions. Data are presented as mean ± SD.

**vRTD**								
	vRTD_25_		vRTD_50_		vRTD_100_		vRTD_MAX_	
	*PLA*	*ALK*	*PLA*	*ALK*	*PLA*	*ALK*	*PLA*	*ALK*
W0	692 ± 557	633 ± 431	1086 ± 762	979 ± 642	1165 ± 547	1097 ± 602	1822 ± 851	1673 ± 878
W5	540 ± 346	420 ± 295	921 ± 587	772 ± 529	1179 ± 578	985 ± 509	1896 ± 904	1589 ± 786
W10	560 ± 301	533 ± 349	909 ± 544	878 ± 577	1218 ± 574	1124 ± 544	2014 ± 901	1754 ± 889
**nRTD**							**MVT**	
	nRTD_25_		nRTD_50_		nRTD_100_			
	*PLA*	*ALK*	*PLA*	*ALK*	*PLA*	*ALK*	*PLA*	*ALK*
W0	2.6 ± 2.2	2.3 ± 1.5	4.1 ± 2.9	3.5 ± 2.1	4.3 ± 1.9	4.2 ± 2.0	6.8 ± 2.9	6.4 ± 3.0
W5	1.9 ± 1.2	1.6 ± 0.9	3.2 ± 1.9	2.9 ± 1.7	4.0 ± 1.6	3.7 ± 1.6	6.4 ± 2.4	6.0 ± 2.4
W10	1.9 ± 1.0	2.0 ± 1.1	3.2 ± 1.9	3.3 ± 1.8	4.3 ± 1.8	4.1 ± 1.9	7.2 ± 2.8	6.5 ± 3.0

### Descending central drive

Voluntary activation (VA; %) was unchanged throughout the training period in either ALK (Wk 0: 87.3 ± 7.2; Wk 10: 87.6 ± 6.7) or PLA (Wk 0: 88.0 ± 6.0; Wk 10: 87.2 ± 1.6). The maximal amplitude of the VL sEMG signal during MVTs (VL_MAX_; %) decreased from Week 0 to Week’s 5 and 10 (Week 5: mean decrease of 2.0 ± 0.6, p < 0.01, η^2^ = 0.78; Week 10: mean decrease of 1.9 ± 0.6, p < 0.05, η^2^ = 0.82), while rates of rise in VL (VL_RER_) increased from Week 0 to Week 10 in the early time periods and again at VL_RERMAX_ (p < 0.05; Table 3). The maximal sEMG amplitude of the VM (VL_MAX_; %) also decreased from Week 0 to Week’s 5 and 10 (Week 5: mean decrease of 3.0 ± 0.8, p < 0.01, η^2^ = 0.81; Week 10: mean decrease of 3.1 ± 0.8, p < 0.01, η^2^ = 0.91). Rates of rise in VM (VM_RER_) did not change with the exception of VM_RER100_ decreasing from Week 0 in both Week’s 5 and 10 (p < 0.05, η^2^ < 0.64; Table 3). In both VL and VM, there was no influence of alkalosis (p > 0.44).

**Table 3:** Presented are the rate of sEMG rise time periods for vastus lateralis (VL) and vastus medialis (VM) (VL_RER_; VL_RER_) normalised to the superimposed M-wave (%) at the beginning (W0), mid-point (W5) and end (W10) of the 10-wk training program for placebo (PLA) and alkalosis (ALK) conditions. Data are presented as mean ± SD.

**VL**								
	VL_RER25_		VL_RER50_		VL_RER100_		VL_RERMAX_	
	*PLA*	*ALK*	*PLA*	*ALK*	*PLA*	*ALK*	*PLA*	*ALK*
W0	38.9 ± 16.0	39.8 ± 18.4	31.8 ± 18.4	28.2 ± 14.0	30.0 ± 15.0	25.6 ± 12.1	100.0 ± 43.2	91.1 ± 36.3
W5	57.5 ± 34.1	62.9 ± 41.6	49.9 ± 24.6*	47.3 ± 24.3*	27.2 ± 13.9	22.0 ± 20.3	129.3 ± 48.4	130.3 ± 79.3
W10	58.2 ± 24.7*	69.6 ± 33.9*	47.3 ± 17.0*	51.6 ± 23.7*	21.0 ± 12.2	19.4 ± 13.7	125.6 ± 30.5*	135.9 ± 61.5*
**VM**								
	VM_RER25_		VM_RER50_		VM_RER100_		VM_RERMAX_	
	*PLA*	*ALK*	*PLA*	*ALK*	*PLA*	*ALK*	*PLA*	*ALK*
W0	42.0 ± 31.2	48.0 ± 24.2	34.2 ± 28.2	36.1 ± 19.7	30.6 ± 17.8	29.6 ± 15.0	140.3 ± 86.3	94.9 ± 68.9
W5	62.9 ± 39.8	50.4 ± 28.6	45.5 ± 21.4	43.0 ± 17.4	22.8 ± 15.3*	17.4 ± 10.3*	140.6 ± 63.8	140.4 ± 74.2
W10	55.1 ± 27.6	50.6 ± 22.0	46.4 ± 18.0	39.9 ± 16.5	17.0 ± 13.2*	14.8 ± 10.1*	134.4 ± 47.0	124.3 ± 42.4

*significantly different from W0 (p < 0.05).

### Evoked contractile properties

Neither maximal resting potentiated twitch amplitude (PT) (p = 0.08), TPT (p = 0.60) nor ^1/2^RT (p = 0.97) changed across the training period for either ALK or PLA (Table 4).

**Table 4:** Presented are the amplitude of the peak resting twitch amplitude (PT; Nm), the time-to-peak twitch (TPT; ms) and 2) the half relaxation time (^1/2^RT; ms) at the beginning (W0), mid-point (W5) and end (W10) of the 10-wk training program for placebo (PLA) and alkalosis (ALK) conditions. Data are presented as mean ± SD.

	PT		TPT		^1/2^RT	
	*PLA*	*ALK*	*PLA*	*ALK*	*PLA*	*ALK*
W0	63.7 ± 15.1	60.1 ± 9.7	73.8 ± 3.5	74.6 ± 6.0	70.1 ± 10.3	72.9 ± 11.8
W5	60.0 ± 13.7	56.5 ± 11.0	75.0 ± 4.0	76.7 ± 5.3	71.6 ± 13.5	67.5 ± 7.0
W10	58.0 ± 11.9	57.1 ± 12.0	75.5 ± 9.6	76.3 ± 7.7	69.9 ± 13.4	71.4 ± 8.1

## Discussion

This investigation aimed to determine whether the recent observations [11,12] that acute NaHCO_3_ supplementation attenuates the reduction in explosive power during fatiguing exercise would manifest into a training adaptation after prolonged (10-wk) training under alkalotic conditions. **Study 1** was conducted in order to confirm acutely whether ingesting 0.3 g·kg^-1^BW NaHCO_3_ would better maintain explosive power after performing a series of leg extension tasks requiring repeated maximal efforts. In this respect, we observed a decline after the exercise task in both maximal torque (MVT) and rates of torque production (RTD) irrespective of acid-base status, however the decline in RTD was attenuated in the alkalotic condition when compared to placebo. The primary finding in **Study 2**, however, would suggest that introducing NaHCO_3_ repeatedly during a 10-week RT program does not confer any additional benefit to the mechanisms (and subsequent adaptive processes) related to explosive power production. Importantly, and with specific reference to applied practice, **Study 2** demonstrates that introducing NaHCO_3_ supplementation twice in a four-day training cycle does not result in any residual or sustained elevation in blood buffering.

The attenuation of explosive torque production but not maximal voluntary force after an acute, fatiguing bout of high-intensity maximal exercise observed in **Study 1** (Fig 3) was similar to previous observations from our laboratory. Briefly, we have demonstrated maximal RTD to be better maintained under alkalosis during fatigue induced by high-power, low force output (i.e. 125% of peak power output) intermittent cycling (30 s work-to-rest ratio) [11]. Also using a dynamic fatiguing model, Higgins and colleagues have shown an attenuating effect of alkalosis on the decline of rapid force generation in mouse fast-twitch fibres [12]. In contrast however, and in both animal and human models, inducing alkalosis has been observed to have little effect on rates of force development when the fatigue is induced through static or isometric involuntary contractions [21,22]. Collectively, this body of work would suggest that at least some degree of repeated, contractile cycling that does not impose significant perfusion limitations on intramuscular blood supply [23] is necessary for NaHCO_3_ to confer a benefit on maintaining RTD during fatiguing exercise. The scale of this interaction between contraction-type and perfusion may also predominate over other fatigue-dependent neural mechanisms linked with a decline in pH (e.g. central drive). Indeed, to date we have only observed NaHCO_3_ to have an effect on measures commensurate with central drive (e.g. VA) during prolonged, ischemic voluntary isometric contractions [24] and not under dynamic conditions such as the present study [25].

Alkalosis had no effect on the number of repetitions performed during the leg extension exercise task (Study 1; Fig 2). This finding is consistent with two other NaHCO_3_ supplementation studies using lower-limb (i.e. leg press), albeit higher % 1-RMs, repeated effort fatiguing tasks [13,14]. Even with longer recovery periods between sets than the present study (30 s vs. > 90 s), neither investigation demonstrated any influence of NaHCO_3_ on the ability to perform work throughout the tasks. Only Coombes and colleagues have observed an ergogenic effect of NaHCO_3_ during both a strength (4-RM maximal leg flexion/extension exercise) and endurance (60 consecutive leg presses at 240°·s^-1^) lower-limb performance task [26]. The authors reported increases in total work (kJ) and peak torque of between 6 and 7%, respectively, during the NaHCO_3_ trials when compared to placebo and control conditions, and attributed this improvement to an increased anaerobic capacity provided by the additional buffer [26]. There are numerous, plausible explanations for the discrepant findings of these studies (e.g. task duration, intensity, total volume of work, etc.), however without a parallel measure of total or accumulated work in the present and formerly discussed studies [13,14], drawing any meaningful explanation without further research is unwarranted.

Improvement in 1-RM leg strength throughout the 10-wk period as a whole demonstrates the efficacy of the RT program (Fig 5), and is consistent with other RT programs conducted over similar time frames with trained participants [27,28]. However we are unable to explain why strength increased owing to the absence of changes in measures derived from the MVCs (Tables 3 and 4), other than the latter testing methodology may not be sensitive enough to detect improvement in trained populations. While studies in novices have reported increased maximal torque and rate of torque with isometric knee extensor testing after dynamic lower limb training [29,30], this has not been replicated in trained populations [28]. It may also be that measurement at the start of the range of motion during leg extension (i.e. 75° knee flexion) is not as sensitive as measurements taken at a more central point of movement, as Mangine and colleagues have recently demonstrated in trained participants using the isometric mid-thigh pull [31].

Although we recognise implementing a balanced training program focused on both power *and* strength may limit specific adaptations associated with rapid force-generating capacity after NaHCO_3_ supplementation (Fig 1), training in such an isolated manner for a period of 10 weeks is unrealistic in any sporting context. Given the limitation of our training program’s multifaceted design (e.g. focus on both strength and power), we would not rule out integrating NaHCO_3_ into various aspects of an RT program. In support, Carr and colleagues have demonstrated that NaHCO_3_ can elicit an improvement in total accumulated training volume (~ 4%) during a single session of whole-body hypertrophy-type resistance training [15]. In a session that was purposefully designed to more than double the total training volume of both the afore mentioned Webster and Portington studies [13,14], these authors attributed the performance improvement to alkalosis attenuating the increased level of physiological strain associated with the higher cumulative load [15]. A reduction in physiological strain during repetitions performed to failure after NaHCO_3_ ingestion has also been observed during a single session, multi-joint resistance exercise (i.e. back squat) [16]. Presumably, the short 30 s rest period between leg extension sets coupled with the repetitions to failure aspect of the present study would have induced a sufficient level of physiological strain and provided an appropriate medium for NaHCO_3_ supplementation. Perhaps introducing a greater total volume or manipulating the training intensity in the current study would have influenced the efficacy of NaHCO_3_.

Finally, the use of NaHCO_3_ on alternate training days did not result in any residual elevation in blood-buffering capacity on the subsequent training day (Fig 4). We believe the thoroughness of the measurements over the 10-wk period may also dispel any myths that beyond acutely (e.g. < 3 or 4 hours) influencing performance outcomes, there is no prolonged ergogenic benefit (e.g. 24 h) from single ingestion protocols. Others have suggested chronic (3 to 5 consecutive days) NaHCO_3_ supplementation of between 0.3 to 0.5 g·kg^-1^BW may result in ergogenic benefits that supersede acute ingestion protocols (e.g. < 180 min prior to exercise) [32,33]. However, McNaughton and colleagues only restricted the final NaHCO_3_ ingestion to 4 hours prior to performing 60 s of intense cycling [33]. As studies have demonstrated since [34,35], blood buffering may still be substantially elevated within this time frame after acute ingestion, thus making it difficult to discern whether the ergogenic benefit observed in the McNaughton study was from the accumulated loading or the acute ingestion prior to the performance task. It is unclear from the methodological description provided by Douroudos and colleagues when the final NaHCO_3_ ingestion took place prior to their 30 s Wingate task [32].

In conclusion, for the first time we have demonstrated that acute ingestion of 0.3 g·kg^-1^BW NaHCO_3_ attenuates the decline of maximal rates of explosive force during a repeated, high-intensity leg extension exercise. However, the differential response observed in **Study 1** did not translate into any further benefit within the neuromuscular mechanisms associated with explosive power production after 10 weeks of training under conditions of metabolic alkalosis. Although 1-RM strength improvements were observed throughout the 10 weeks, these improvements were independent of acid-base balance. Finally, given the efficacy of NaHCO_3_ supplementation may be influenced by the relationship between training volume and intensity [15,16], future work should build upon the findings of this initial 10-wk training study to further elucidate whether or not integrating NaHCO_3_ supplementation into a resistance-training program is warranted.

## Supporting information

S1 Dataset Supplemental raw data is available for this study. (ZIP)
